# Polypill eligibility and equivalent intake in a Swiss population-based study

**DOI:** 10.1038/s41598-021-84455-8

**Published:** 2021-03-25

**Authors:** Julien Castioni, Nazanin Abolhassani, Peter Vollenweider, Gérard Waeber, Pedro Marques-Vidal

**Affiliations:** Department of Medicine, Internal Medicine, Lausanne University Hospital and University of Lausanne, Lausanne, Switzerland

**Keywords:** Epidemiology, Clinical pharmacology

## Abstract

The polypill has been advocated for cardiovascular disease (CVD) management. The fraction of the population who could benefit from the polypill in Switzerland is unknown. Assess (1) the prevalence of subjects (a) eligible for the polypill and (b) already taking a polypill equivalent; and (2) the determinants of polypill intake in the first (2009–2012) and second follow-ups (2014–2017) of a population-based prospective study conducted in Lausanne, Switzerland. The first and the second follow-ups included 5038 and 4596 participants aged 40–80 years, respectively. Polypill eligibility was defined as having a high CVD risk as assessed by an absolute CVD risk ≥ 5% with the SCORE equation for Switzerland and/or presenting with CVD. Four polypill equivalents were defined: statin + any antihypertensive with (A) or without (B) aspirin; statin + calcium channel blocker (CCB) (C); and statin + CCB + angiotensin-converting enzyme inhibitor (D). The prevalence of polypill eligibility was 20.6% (95% CI 19.5–21.8) and 27.7% (26.5–29.1) in the first and second follow-up, respectively. However, only around one-third of the eligible 29.5% (95% CI 26.7–32.3) and 30.4% (27.9–33.0) respectively, already took the polypill equivalents. All polypill equivalents were more prevalent among men, elderly and in presence of CVD. After multivariable adjustment, in both periods, male gender was associated with taking polypill equivalent A (OR: 1.93; 95% CI 1.45–2.55 and OR: 1.67; 95% CI 1.27–2.19, respectively) and polypill equivalent B (OR: 1.52; 95% CI 1.17–1.96 and OR: 1.41; 95% CI 1.07–1.85, respectively). Similarly, in both periods, age over 70 years, compared to middle-age, was associated with taking polypill equivalent A (OR: 11.71; CI 6.74–20.33 and OR: 9.56; CI 4.13–22.13, respectively) and equivalent B (OR: 13.22; CI 7.27–24.07 and OR: 20.63; CI 6.51–56.36, respectively). Former or current smoking was also associated with a higher likelihood of taking polypill equivalent A in both periods. A large fraction of the population is eligible for the polypill, but only one-third of them actually benefits from an equivalent, and this proportion did not change over time.

## Introduction

Cardiovascular disease (CVD) is the major cause of death and disability worldwide^1^. The reduction of CVD burden requires strategies that are applied to the entire or to large segments of the population^2^. Despite effective cardiovascular drugs, adherence is suboptimal in both primary and secondary prevention^3^. The “polypill”, a fixed dose combination of statin, one or more anti-hypertensive drugs with or without aspirin, could increase adherence^4,5^ and reduce CVD burden^6,7^. Several strategies have been proposed for using the polypill: mass treatment for all elderly subjects^8,9^; primary prevention among subjects at high CVD risk^10^, and secondary prevention among patients with established CVD^11^. Which strategy is the best is still under debate^12^. Currently, it is estimated that a sizable fraction of the general population is already taking the polypill components as separate drugs. Replacing these separate drugs by a fixed dose combination (polypill) could improve adherence to treatment and reduce CVD. There are different types of polypill equivalents available^13,14^, but the fraction of the population who could (1) benefit from the polypill or (2) who is already taking the polypill components (i.e., a polypill equivalent) as separate drugs is largely unknown.

This study aimed to assess (1) the prevalence of subjects (a) eligible for the polypill and (b) already taking a polypill equivalent; and (2) the determinants of polypill intake in the first (2009–2012) and second follow-up (2014–2017) of a prospective population-based study conducted in Lausanne, Switzerland. We also assessed which components of the different polypills were already prescribed.

## Materials and methods

### Study population and design

The Colaus/PsyColaus study (http://www.colaus-psycolaus.ch) is an ongoing prospective survey investigating the biological and genetic determinants of CV risk factors and CVD in the population of Lausanne, Switzerland. Detailed descriptions of the study design have been reported elsewhere^15^. In summary, a non-stratified random sample of the overall population of Lausanne aged between 35 and 75 years was drawn. The following inclusion criteria were applied: (a) written informed consent; (b) willingness to take part in the examination and to provide blood samples. Recruitment began in June 2003 and ended in May 2006 and included 6733 participants. Participation rate was 41%. The evaluation included an interview, a physical exam, blood sampling and a set of questionnaires.

The follow-up visit was similar to the baseline evaluation and was performed between April 2009 and September 2012, five and a half years on average after the collection of baseline data, and 5064 subjects participated; out of them, 5038 (99.5%) who had CVD risk data were included in this study. Similarly, for the second follow up (2014–2017), 4881 subjects participated; out of them 4596 (94.2%) who had CVD risk data were included in the study. The data from the first and second follow-ups were used in this study (Supplemental Figure 1, panels A and B, respectively).

### Data collected

CVD, lifestyle and medication status were assessed by questionnaire. CVD was defined as personal history of myocardial infarction (MI), unstable or stable angina, coronary artery bypass grafting, stroke, transient ischemic attack or peripheral arterial disease. Whenever possible, CVD reported by participants was documented and adjudicated by an independent panel of experts. Smoking status was categorized into never, former and current as reported. Medications were assessed by questionnaire and further confirmed by interview, where subjects were asked to provide the names of all prescribed and over-the-counter drugs regularly taken over the last 6 months. No specific information was collected whether it was the general practitioner or the cardiologist who prescribed the medications.

Polypill eligibility was defined as presenting with previous CVD (secondary prevention) or, in the absence of CVD, an absolute CVD risk ≥ 5% assessed by the SCORE equation for Switzerland^16^ (primary prevention). Four commercially available polypill equivalents were defined: equivalent A: aspirin + statin + any antihypertensive; equivalent B: no aspirin + statin + any antihypertensive; equivalent C: no aspirin + statin + calcium channel blocker (equivalent to Caduet in Switzerland); equivalent D: no aspirin + statin + calcium channel blocker + angiotensin-converting enzyme inhibitor (equivalent to Triveram in Switzerland). The last two are the only approved polypills in Switzerland.

### Statistical analysis

Statistical analyses were conducted separately for each survey period (2009–2012 and 2014–2017). Analyses were performed using STATA software 16.0 (Stata Corp, College Station, TX, USA). Descriptive results were expressed as number of participants (percentage) for all categorical variables. Exact Poisson confidence intervals for prevalence rates for polypill eligibility and for each polypill equivalent were obtained using the ci proportions command of Stata. Bivariate analysis comparing the distribution of the different covariates (i.e. gender, age group…) within each polypill equivalent (use/non-use) was performed using chi-square or Fisher’s exact test. Multivariate analysis assessing which covariates were associated with use of each polypill equivalent was conducted using logistic regression with the polypill equivalent (use/non-use) as dependent variable and gender (man, woman), age group ([40–50], [50–60], [60–70], [70+]), smoking status (never, former, current) and history of CVD (yes, no) as independent variables. Results were expressed as odds ratio (OR) and 95% confidence interval (CI). A two-sided test p-value below 0.05 was considered statistically significant.

### Ethical statement

The institutional Ethics Committee of the University of Lausanne, which afterwards became the Ethics Commission of Canton Vaud (http://www.cer-vd.ch) approved the baseline CoLaus study (reference 16/03). The approval was renewed for the first (reference 33/09) and the second (reference 26/14) follow-ups. The study was performed in agreement with the Helsinki declaration and its former amendments, and in accordance with the applicable Swiss legislation. Written informed consent was obtained from all participants.

## Results

### Characteristics of the participants

The characteristics of the included and excluded participants are summarized in supplemental Table 1. In the first follow-up, of the 5064 participants available, 26 (0.5%) were excluded; no differences were found between included and excluded participants. In the second follow-up, of the 4881 participants available, 285 (5.8%) were excluded; excluded participants were more frequently in the age group [60–70] and less frequently former smokers than included participants.

### Prevalence of polypill eligibility and polypill equivalents

The distribution of eligibility and any polypill equivalents intake are summarized in supplemental Table 2. Prevalence of polypill (primary and secondary) eligibility and of the different polypill equivalents is summarized in Table 1. The prevalence of polypill eligibility, based on previous CVD or a CVD SCORE risk ≥ 5%, increased from one-fifth in the first follow-up to over one quarter of participants in the second. The majority of eligible subjects were in the primary prevention arm. The prevalence of eligible subjects taking polypill components was lower and around one-third of the eligible subjects in the first and second follow-up. Polypill equivalent A had the highest prevalence in both periods, followed by B, C and D.

**Table 1:** Prevalence of polypill eligibility and of the different polypill equivalents, Colaus study, Lausanne, Switzerland, 2009–2012 and 2014–2017.

	2009–2012	2014–2017
**Polypill eligibility**		
All	20.6 (19.5–21.8)	27.7 (26.5–29.1)
Primary prevention	12.8 (11.9–13.7)	16.5 (15.4–17.6)
Secondary prevention	7.8 (7.1–8.6)	11.3 (10.4–12.2)
**Polypill equivalents intake among eligible**		
Any	29.5 (26.7–32.3)	30.4 (27.9–33.0)
Equivalent A^a^	19.7 (17.4–22.3)	19.0 (16.9–21.2)
Equivalent B^b^	9.7 (8.0–11.7)	11.5 (9.8–13.3)
Equivalent C^c^	6.6 (5.2–8.3)	7.4 (6.0–8.9)
Equivalent D^d^	2.6 (1.7–3.8)	2.0 (1.3–2.9)

Results are expressed as percentage and 95% confidence interval.

^a^Aspirin + statin + any antihypertensive.

^b^No aspirin + statin + any antihypertensive.

^c^No aspirin + statin + calcium channel blocker (equivalent to Caduet in Switzerland).

^d^No aspirin + statin + calcium channel blocker + angiotensin-converting enzyme inhibitor (equivalent to Triveram in Switzerland).

### Determinants of polypill equivalent use

The bivariable and multivariable associations between selected sociodemographic and clinical variables and the type of polypill equivalent used are summarized in Tables 2 and 3, respectively. Irrespective of the type of polypill equivalent, in both periods, the prevalence was higher among men, the elderly and participants with a history of CVD (Table 2). After multivariable adjustment, male gender, increasing age or history of CVD were associated with a higher likelihood of taking polypill equivalents A or B in both periods, and former or current smokers with a higher likelihood of taking polypill equivalent A in both periods (Table 3).

**Table 2:** bivariate associations between selected clinical variables and type of polypill equivalent, CoLaus study, Lausanne, Switzerland, 2009–2012 and 2014–2017.

	2009–2012				2014–2017			
	A	B	C	D	A	B	C	D
**Gender**								
Woman	97 (3.6)	119 (4.4)	46 (1.7)	12 (0.5)	119 (4.7)	128 (5.1)	59 (2.3)	15 (0.6)
Man	182 (7.8)	144 (6.1)	81 (3.5)	28 (1.2)	204 (9.9)	138 (6.7)	84 (4.1)	22 (1.1)
p-value	< 0.001	0.006	< 0.001	0.003	< 0.001	0.021	0.001	0.077
**Age group**								
[40–50]	16 (1.1)	13 (0.9)	9 (0.6)	3 (0.2)	6 (1.1)	3 (0.5)	1 (0.2)	1 (0.2)
[50–60]	44 (2.9)	50 (3.3)	25 (1.6)	8 (0.5)	37 (2.5)	38 (2.6)	11 (0.8)	1 (0.1)
[60–70]	101 (7.5)	118 (8.8)	43 (3.2)	8 (0.6)	83 (6.8)	74 (6.1)	34 (2.8)	10 (0.8)
[70+]	118 (16.0)	82 (11.1)	50 (6.8)	21 (2.9)	197 (14.7)	151 (11.3)	97 (7.2)	25 (1.9)
p-value	< 0.001	< 0.001	< 0.001	< 0.001^§^	< 0.001	< 0.001	< 0.001	< 0.001^§^
**Smoking status**								
Never	72 (3.6)	103 (5.1)	40 (2.0)	8 (0.4)	81 (4.6)	107 (6.0)	46 (2.6)	11 (0.6)
Former	155 (8.3)	113 (6.0)	62 (3.3)	23 (1.2)	158 (9.3)	99 (5.9)	57 (3.4)	16 (1.0)
Current	51 (4.7)	47 (4.3)	25 (2.3)	9 (0.8)	61 (7.7)	38 (4.8)	25 (3.1)	5 (0.6)
p-value	< 0.001	0.125	0.026	0.014	< 0.001	0.430	0.386	0.483
**History of CVD**								
No	192 (3.9)	242 (5.0)	97 (2.0)	29 (0.6)	173 (4.1)	217 (5.1)	103 (2.4)	21 (0.5)
Yes	87 (52.7)	21 (12.7)	30 (18.2)	11 (6.7)	150 (43.6)	49 (14.2)	40 (11.6)	16 (4.7)
p-value	< 0.001	< 0.001	< 0.001	< 0.001	< 0.001	< 0.001	< 0.001	< 0.001

Results are expressed as number (row percentage). Equivalent A: aspirin + statin + any antihypertensive; equivalent B: no aspirin + statin + any antihypertensive; equivalent C: no aspirin + statin + calcium channel blocker; equivalent D: no aspirin + statin + calcium channel blocker + angiotensin-converting enzyme inhibitor. Between-group comparisons performed using chi-square or Fisher’s exact test (§).

**Table 3:** Multivariable association between selected clinical variables and type of polypill equivalent, CoLaus study, Lausanne, Switzerland, 2009–2012 and 2014–2017.

	2009–2012		2014–2017	
	Polypill equivalent A	Polypill equivalent B	Polypill equivalent A	Polypill equivalent B
**Gender**				
Woman	1 (ref.)	1 (ref.)	1 (ref.)	1 (ref.)
Man	**1.93 (1.45–2.55)**	**1.52 (1.17–1.96)**	**1.67 (1.27–2.19)**	**1.41 (1.07–1.85)**
**Age group**				
[40–50]	1 (ref.)	1 (ref.)	1 (ref.)	1 (ref.)
[50–60]	**2.27 (1.26–4.08)**	**3.64 (1.97–6.74)**	1.91 (0.79–4.62)	**4.76 (1.46–15.50)**
[60–70]	**5.83 (3.37–10.06)**	**10.48 (5.87–18.72)**	**4.76 (2.03–11.17)**	**11.15 (3.49–35.64)**
[70+]	**11.71 (6.74–20.33)**	**13.22 (7.27–24.07)**	**9.56 (4.13–22.13)**	**20.63 (6.51–65.36)**
p-value for trend	< 0.001	< 0.001	< 0.001	< 0.001
**Smoking status**				
Never	1 (ref.)	1 (ref.)	1 (ref.)	1 (ref.)
Former	**1.86 (1.35–2.55)**	0.99 (0.74–1.31)	**1.69 (1.24–2.31)**	0.81 (0.60–1.08)
Current	**1.60 (1.10–2.39)**	0.98 (0.68–1.41)	**2.11 (1.43–3.10)**	0.89 (0.60–1.31)
p-value for trend	0.023	0.920	< 0.001	0.549
**History of CVD**				
No	1 (ref.)	1 (ref.)	1 (ref.)	1 (ref.)
Yes	**14.65 (10.21–21.02)**	1.50 (0.92–2.46)	**12.34 (9.23–16.50)**	**1.93 (1.33–2.78)**

Results are expressed as odds ratio (95% confidence interval). Equivalent A: aspirin + statin + any antihypertensive; equivalent B: no aspirin + statin + any antihypertensive. Statistical analysis using logistic regression. Significant (p < 0.05) odds ratios are indicated in bold.

### Distribution of polypill components

The distribution of polypill components is summarized in supplemental Table 3. The most prescribed component was antihypertensive drugs, of which the most prescribed were angiotensin receptor blockers, followed by beta-blockers. Statins were the most frequent prescribed hypolipidaemic drug and aspirin was the most frequently prescribed antiplatelet drug.

## Discussion

In this population-based study, over one-fifth and one-fourth of the participants were eligible for a polypill in two time points 5 years apart. Nevertheless, only one-third of them actually benefits from an equivalent, and this proportion did not change over time. Male gender, increasing age or history of CVD were associated with a higher likelihood of taking a polypill equivalent.

### Prevalence of polypill eligibility and polypill intake

The eligibility for polypill is a matter of debate, especially in primary prevention, and can explain the low prevalence of polypill equivalent in our study in comparison to eligible subjects, based on the SCORE equation for Switzerland and previous CVD^16^. In comparison, the intake of polypill components was also low in a study in the US, which considered age ≥ 55 years and a history of CVD as polypill eligibility criteria. They showed that 37.3% of US adults ≥ 55 years and 57.0% of those with a history of CVD were taking statins and the use of other polypill medications was also low^17^. Such results can be comparable with ours and the higher intake of polypill can be due to the different eligibility criteria. The difference between use of polypill in secondary, compared to the primary prevention, was lower which can be due to the broader support for the use of polypill in secondary prevention of CVD^18^. Our results imply a gap between an individual cardiovascular risk target approach and a population-based global strategy that should co-exist to tackle the burden of CVD.

Although polypills have been approved in more than 30 countries and the effectiveness in preventing major CV events, increasing medication adherence and lowering number of adverse events has been reported, their availability remains limited^13,19^. A large availability, with many types and doses combination, is essential for polypill in primary high risk and secondary prevention as demonstrated in an Australian study, where, even if 62.4% of the patients were still taking the polypill after 18 months, 47.4% of them had additional classes of antihypertensive drugs prescribed^5^. Even considering only fixed drug combination for antihypertensive therapy, availability remains poor. For example, in an American study, the most frequently filled fixed drug combination lisinopril/HCTZ was marketed only in 3 doses, although 20 combinations are possible^21^. In the Swiss market, no polypill with aspirin exists although more than one out of 20 participants could benefit from it, and only two equivalents without aspirin exist (C and D). This underlines the potential benefits of a larger availability of a polypill in Switzerland to improve adherence in primary or secondary preventive approach.

### Determinants of polypill equivalents intake

Male gender, increased age and previous history of CVD were associated with higher frequency of the polypill equivalents intake; this observation is consistent with gender and age differences in CVD risk factors^16,20^. For polypill equivalent A (aspirin + statin + any hypertensive drugs), also a positive association with smoking and previous history of CVD was found in both first and second follow-ups. For polypill equivalent B (no aspirin + statin + any hypertensive drugs), the association with previous history of CVD was only significant in the second follow-up.

### Prevalence of polypill components

Antihypertensive drugs are the most frequently prescribed polypill component (one quarter of the subjects). This could reflect the high proportion of hypertension in the Swiss population, estimated as 36%^21^, and a high adherence to hypertension target treatment recommendations^22^. Regarding antihypertensive drug treatment, studies showed polypills containing multiple low doses of BP-lowering drugs produce more effective BP lowering than the use of fewer separate BP-lowering drugs at higher doses, without an increase in adverse effects^7,18^. In our study, angiotensin receptor blockers were the most used hypertensive drug class. It is quite different from other studies where ACEI inhibitors were the most frequently prescribed class, as shown in an Australian study comparing the adherence of high risk and established CV patients to a polypill group (simvastatin, lisinopril and atenolol or HCTZ) vs standard medication group^5^. In this study, all patients were treated by one of the 38 different blood pressure-lowering medications. ACE inhibitors are the most prescribed followed by BB and then ARB. In USA, Wang et al. examined prescriptions fill rates for dyslipidemia, diabetes and hypertension treatments using claims data from a large national insurer for > 14 million people^23^. The most prescribed combination was a statin and hypertensive molecule of which ACEi was the most frequent, followed by BB, CCB and ARB. The switch between ACEi and ARB between our study and two others could be explained by the higher risk profile of the subjects in the mentioned studies. Also, easy access to and marketing of ARB in Switzerland could play a role. Aspirin is much more frequent than other antiplatelet medications as it is the only one to be indicated in primary prevention. Statins are by far the most prescribed hypolipidaemic drug class according to the Swiss atherosclerosis association Swiss recommendations (http://www.gsla.ch). Nevertheless, another study in Switzerland also showed no parallel increase in hypolipidemic drugs along with increased dyslipidaemia prevalence and its management remained far from optimal in both primary and secondary prevention^24^. Also, a study in 78 centers from 16 European countries revealed that large proportions of people at high CVD risk have inadequate control of blood pressure, lipids and diabetes^25^. Suboptimal management of coronary patients was reported in a 24 European countries in 2015^26^ and further in 27 European countries in 2019^27^. In addition, there are differences in guidelines of the use of statins in primary prevention in Europe and other countries that could also contribute to the low use of this drug class in this indication^28,29^.

### Strengths and limitations

This study evaluates polypill eligibility in a general population sample of more than 6000 persons. Contrary to the polypill eligibility, we assessed the real life intake of polypill equivalents, defined by the intake of separated components of polypill, and its change in five years.

As limitations, we could cite the selection bias of the general population, described elsewhere^15^. In addition, as drugs data were self-reported, it might be prone to recall bias and finally underestimation. However, our results can provide at least an estimation of the real life polypill intake and of the gap to the theoretic eligibility. The results should be interpreted cautiously due to the certain criteria for eligibility that we used in this study i.e. having a high CVD risk and/or presenting with CVD, could be different in other countries and even largely underestimated if following mass treatment criteria as suggested by Wald and colleagues^9^. Using this criterion such as age  + 50 years, the theoretic eligibility would be 72% and 88% in the two follow-ups, respectively, and the gap with real life even larger as only about one-seventh of them already taking polypill equivalents at both periods. In addition, we used SCORE for CVF risk stratification, however, GSLA score may be also used in Switzerland. Furthermore, this was a monocentric study and might not be fully generalizable to other places with different health system and availability of polypills. Even, within a country like Switzerland, different prescription prevalence across cantons has been reported^30^ so generalizability should be considered given such limitations.

## Conclusion

In this prospective study in Swiss community-dwelling subjects, over one-fifth and one-fourth were eligible for a polypill. Nevertheless, only one-third of them actually benefits from an equivalent, and this proportion did not change over time.

## Supplementary Information

Supplementary Information 1.

Supplementary Information 2.

## Abbreviations

MI: Myocardial infarction
ACEi: Angiotensin converting enzyme inhibitors
ARB: Angiotensin receptors blockers
CCB: Calcium-channel blockers
BB: Beta-blockers
antiHTA: Antihypertensive
CVD: Cardiovascular disease
FDC: Fixed dose combination
