# Supplementary material for: Polypill eligibility and equivalent intake in a Swiss population-based study
Source: Sci Rep. 2021 Mar 25;11:6880. doi: 10.1038/s41598-021-84455-8 (PMC7994372; doi:10.1038/s41598-021-84455-8)
Supplement: Supplementary file 1 — Supplementary Information 1. [file 41598_2021_84455_MOESM1_ESM.pptx]

## Slide 1
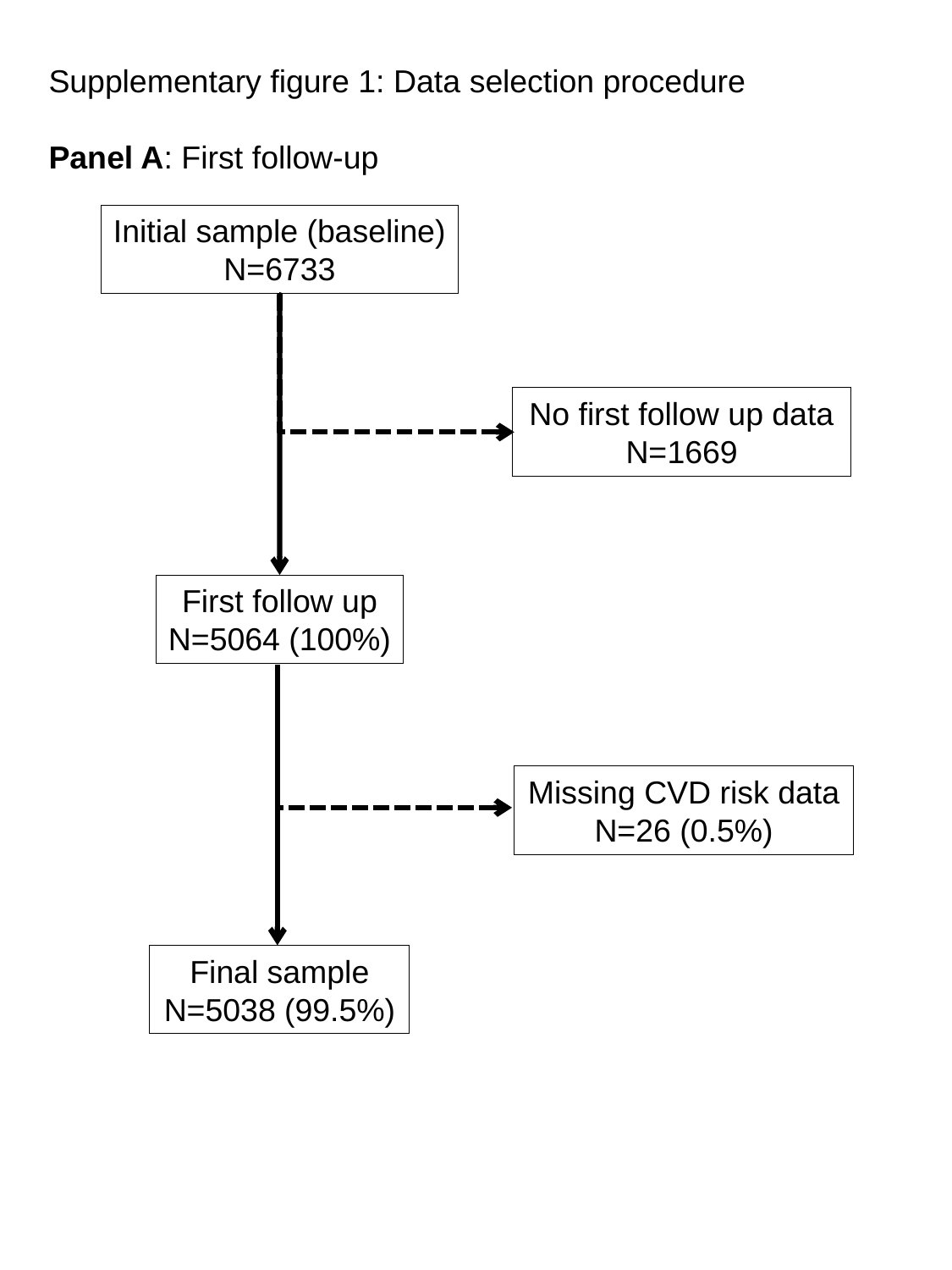

Supplementary figure 1: Data selection procedure
Panel A: First follow-up
Initial sample (baseline)
N=6733
No first follow up data N=1669
First follow up
N=5064 (100%)
Missing CVD risk data N=26 (0.5%)
Final sample
N=5038 (99.5%)

## Slide 2
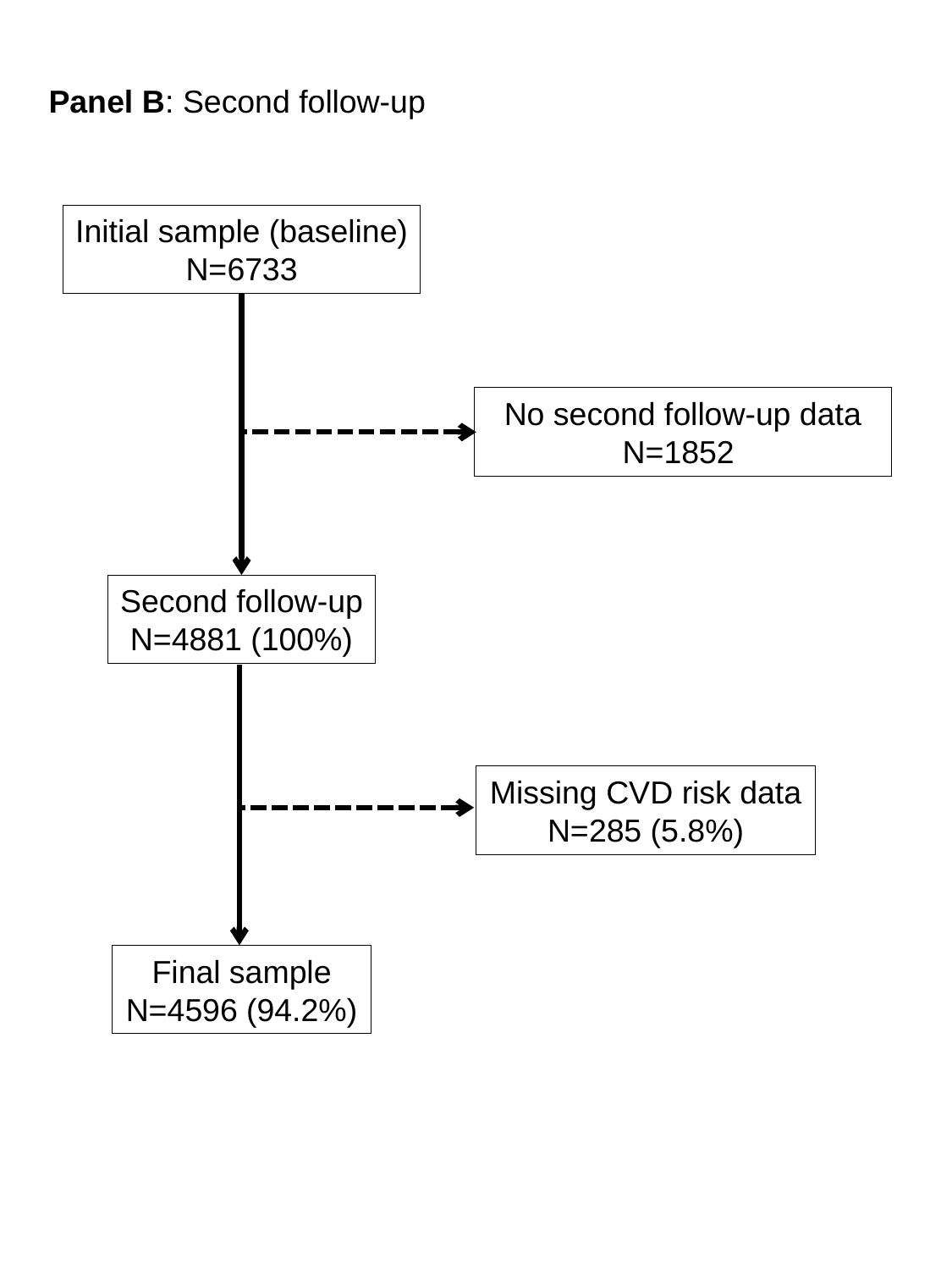

Panel B: Second follow-up
Initial sample (baseline)
N=6733
No second follow-up data N=1852
Second follow-up
N=4881 (100%)
Missing CVD risk data N=285 (5.8%)
Final sample
N=4596 (94.2%)
