# Supplementary material for: Polypill eligibility and equivalent intake in a Swiss population-based study
Source: Sci Rep. 2021 Mar 25;11:6880. doi: 10.1038/s41598-021-84455-8 (PMC7994372; doi:10.1038/s41598-021-84455-8)
Supplement: Supplementary file 2 — Supplementary Information 2. [file 41598_2021_84455_MOESM2_ESM.docx]

**POLYPILL ELIGIBILITY AND EQUIVALENT INTAKE IN A SWISS POPULATION-BASED STUDY**

Julien Castioni*, MD; Nazanin Abolhassani*, PhD; Peter Vollenweider; Gérard Waeber, MD; Pedro Marques-Vidal, MD, PhD.

Department of Medicine, Internal Medicine, Lausanne university hospital and University of Lausanne, Lausanne, Switzerland

* These authors contributed equally

**Authors’ emails:**

Julien Castioni: Julien.Castioni@chuv.ch

Nazanin Abolhassani: [Nazanin.abolhassani@chuv.ch](mailto:na_abolhassani@yahoo.com)

Peter Vollenweider: Peter.Vollenweider@chuv.ch

Gérard Waeber: Gerard.Waeber@chuv.ch

Pedro Marques-Vidal: [Pedro-Manuel.Marques-Vidal@chuv.ch](mailto:Pedro-Manuel.Marques-Vidal@chuv.ch)

**Authors’ ORCID**

Julien Castioni: [0000-0002-7260-9371](https://orcid.org/0000-0002-7260-9371)

Nazanin Abolhassani: 0000-0002-3616-3595

Peter Vollenweider: 0000-0002-0765-896X

Gérard Waeber: 0000-0003-4193-788X

Pedro Marques-Vidal: 0000-0002-4548-8500

**Address for correspondence and reprints**

Nazanin Abolhassani

Room BH10-636

Department of Medicine, Internal Medicine

Lausanne university hospital

Rue du Bugnon 46

1011 Lausanne

Switzerland

Phone: +41 21 314 09 82

Email: Nazanin.abolhassani@chuv.ch

**SUPPLEMENTAL TABLES**

**Supplemental table 1:** characteristics of included and excluded participants, Colaus study, Lausanne, Switzerland, 2009-2012 and 2014-2017.

|  | **First follow-up (2009-2012) (N=5064)** | | | **Second follow-up (2014-2017) (N=4881)** | | |
| --- | --- | --- | --- | --- | --- | --- |
|  | **Included** | **Excluded** | **P-value** | **Included** | **Excluded** | **P-value** |
| All | 5038 (99.5) | 26 (0.5) |  | 4596 (94.2) | 285 (5.8) |  |
| Gender |  |  | 0.968 |  |  | 0.462 |
| Woman | 2693 (53.5) | 14 (53.8) |  | 2526 (55.0) | 163 (57.2) |  |
| Man | 2345 (46.5) | 12 (46.2) |  | 2070 (45.0) | 122 (42.8) |  |
| Age group |  |  | 0.653 |  |  | 0.002 |
| [40-50[ | 1421 (28.2) | 10 (38.5) |  | 561 (12.2) | 19 (6.7) |  |
| [50-60[ | 1533 (30.4) | 7 (26.9) |  | 1474 (32.1) | 96 (33.7) |  |
| [60-70[ | 1348 (26.8) | 5 (19.2) |  | 1222 (26.6) | 99 (34.7) |  |
| [70+ | 736 (14.6) | 4 (15.4) |  | 1339 (29.1) | 71 (24.9) |  |
| Smoking status |  |  | 0.813 |  |  | 0.001 |
| Never | 2025 (40.7) | 10 (38.5) |  | 1781 (41.7) | 105 (46.9) |  |
| Former | 1874 (37.6) | 9 (34.6) |  | 1692 (39.6) | 62 (27.7) |  |
| Current | 1082 (21.7) | 7 (26.9) |  | 798 (18.7) | 57 (25.4) |  |
| History of CVD |  |  | 0.422 § |  |  | <0.001 § |
| No | 4873 (96.7) | 26 (100.0) |  | 4252 (92.5) | 285 (100.0) |  |
| Yes | 165 (3.3) | 0 (0.0) |  | 344 (7.5) | 0 (0.0) |  |

Results are expressed as number (percentage). Between-group comparisons performed using chi-square or Fisher’s exact test (§).

**Supplemental table 2:** Prevalence of polypill eligibility and any polypill equivalents intake, Colaus study, Lausanne, Switzerland, 2009-2012 and 2014-2017.

|  | Eligible, 2009-2012 | | Eligible, 2014-2017 | |
| --- | --- | --- | --- | --- |
| Any polypill equivalent intake | No | Yes | No | Yes |
| No | 3120 | 887 | 3763 | 733 |
| Yes | 201 | 388 | 236 | 306 |

**Supplemental table 3**: Drugs prescribed, Colaus study, Lausanne, Switzerland, 2009-2012 and 2014-2017.

| **Prescribed Drugs** | **2009-2012 (N=5038)** | **2014-2017 (N=4596)** |
| --- | --- | --- |
| Antiplatelet drugs |  |  |
| Any | 585 (11.6) | 699 (12.8) |
| Aspirin | 559 (11.1) | 655 (14.3) |
| Hypolipidaemic drug treatment |  |  |
| Any | 932 (18.5) | 936 (20.4) |
| Statins | 880 (17.5) | 876 (19.1) |
| Other hypolipidemic drugs | 79 (1.6) | 91 (2.0) |
| Antihypertensive drug treatment |  |  |
| Any | 1352 (26.3) | 1525 (33.2) |
| Angiotensin receptor blockers | 654 (13.0) | 765 (16.6) |
| ACE inhibitors | 358 (7.1) | 408 (8.9) |
| Beta-blockers | 452 (9.0) | 525 (11.4) |
| Calcium channel blockers | 271 (5.4) | 328 (7.1) |
| Diuretics | 405 (8.0) | 491 (10.7) |
| Hydrochlorothiazide | 259 (5.1) | 333 (7.3) |
| Other antihypertensive drugs § | 18 (0.4) | 25 (0.5) |

Results are expressed as number of participants (percentage). § peripheral vasodilators or post-synaptic stimulators.
